# Extensive permethrin and DDT resistance in *Anopheles arabiensis *from eastern and central Sudan

**DOI:** 10.1186/1756-3305-4-154

**Published:** 2011-08-03

**Authors:** Yousif E Himeidan, Hamid M Abdel Muzamil, Christopher M Jones, Hilary Ranson

**Affiliations:** 1 Entomology Unit, Faculty of Agriculture and Natural Resources, University of Kassala, New Halfa, Sudan; 2 Institute of Endemic Diseases (IEND), University of Khartoum; 3 Vector Group, Liverpool School of Tropical Medicine, Pembroke Place, Liverpool, UK, L3 5QA

## Abstract

**Background:**

The distribution of insecticide treated nets (ITN) has been dramatically scaled up in eastern and central Sudan. Resistance to insecticides has already been reported in this region and there is an urgent need to develop appropriate resistance management strategies, which requires detailed information on the extent and causes of resistance. This study assessed resistance to permethrin and DDT in seven populations of *Anopheles arabiensis *from Sudan.

**Results:**

Three out of the seven populations were defined as resistant to permethrin and five of six populations resistant to DDT according to WHO criteria. The 1014F kdr allele was present in all six populations tested and the presence of this allele was significantly correlated with resistance to permethrin (*P *= 0.0460). While homozygous 1014F individuals were statistically not more likely to survive (53.7%) permethrin than to be killed (38.6%) by the diagnostic dose, there was no difference in the likelihood of permethrin survival in heterozygotes (*P *= 0.7973). The susceptible genotypes were more likely to be killed by permethrin exposure than to survive (*P *= 0.0460). The 1014F allele failed to confer a survival advantage to the WHO diagnostic dose of DDT in either the homozygous or heterozygous state. The 1014S allele was not detected in any of the populations tested.

**Conclusion:**

The kdr allele is certainly contributing to the extensive resistance to permethrin and DDT in Sudan but the high number of DDT (43%) and permethrin (16.7%) survivors that did not contain either kdr alleles suggests that other resistance mechanisms are also present in these populations. The high frequency of permethrin resistance throughout central and eastern Sudan is a cause of great concern for malaria control activities.

## Background

Ongoing strategies of malaria vector control rely greatly on the use of indoor residual spraying (IRS) and insecticide-treated nets (ITNs). The current success of these strategies in reducing malaria contributed towards the optimism that elimination of this disease as a public health problem is a feasible objective [1]. Substantial international efforts have been made during the last three years enabling access to approximately 289 million ITNs in sub-Saharan Africa, enough to cover 76% of the 765 million people at risk of malaria. The number of countries that employed IRS as vector control strategy increased from 31 in 2007 to 68 in 2009 [2]. Further scale- up of IRS and ITNs is occurring throughout the African continent.

ITNs and, to a large extent, IRS are highly dependent on pyrethroid insecticides. The widespread use of this class of insecticide increases the risk of resistance. The situation may be accelerated by the reintroduction of DDT in several countries in Africa as cross-resistance between these insecticide classes can occur as a result of amino acid substitutions in the shared target site. All major malaria vectors in Africa have developed resistance to these insecticides and the resistance alleles appear to be spreading at an exceptionally rapid rate [3].

Pyrethroids and DDT target the voltage-gated sodium channel site. Two alternative substitutions of the leucine 1014 residue can lead to target site resistance. The 1014F allele was first identified in strains of *An. gambiae *from Burkina Faso and Côte d'Ivoire [4] and the 1014S allele was later identified in this species in Kenya [5]. Both alleles are now widely distributed in *An. gambiae *[3]. In *An. arabiensis*, 1014F has been found in several widely dispersed populations from Burkina Faso [6,7], Tanzania [8], Sudan [9,10], Senegal [11] and Ethiopia [12,13]. The 1014S allele was also observed in wild populations of *An. arabiensis *from Uganda [14] and western Kenya [15,16]. Both 1014F and 1014S alleles have been detected together in the same populations in Sudan [9] and Cameroon [17].

In Sudan, our surveys in 2005 showed that the frequency of the 1014F allele in *An. arabiensis *was more than double in areas where insecticide-treated nets were used compared to a cotton growing area which was regularly treated with insecticides [9]. The result suggested that pyrethroid-based vector control may extend and increase kdr distribution. The distribution of ITNs in Sudan has been scaled up dramatically in recent years and 60% ITN coverage rate has been achieved (National Malaria Control Programme, unpublished data). There is therefore an urgent need to monitor the distribution of resistance and to develop appropriate resistance management strategies. The data presented will further assist in this process.

## Methods

### Study sites

**Figure 1:**
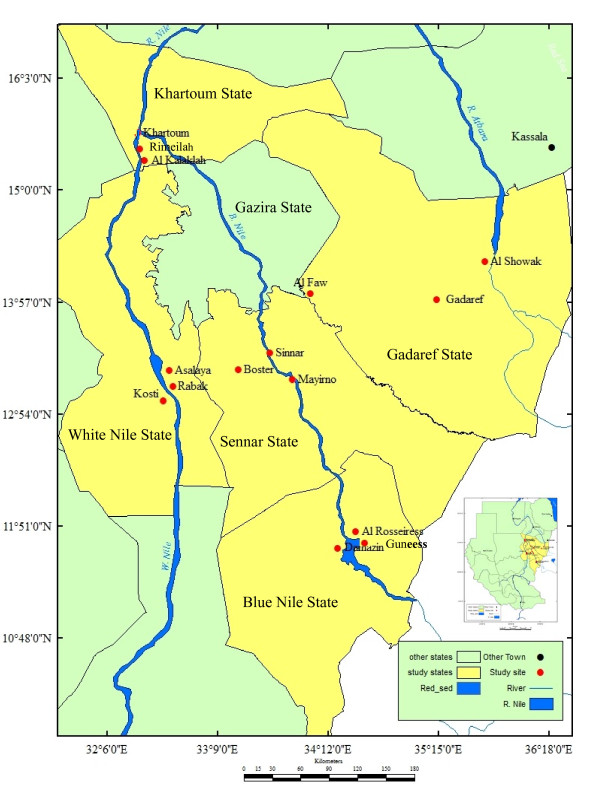
**Map showing the study sites in Central and Eastern Sudan**.

The study was carried out in five states of eastern and central Sudan. The states surveyed were Sennar [sites surveyed were Sennar town (33° 55' E, 13° 10' N), Al Boster (33° 36' E, 13° 32' N) and Mayirno (33° 66' E, 13° 47' N)], Blue Nile [site surveyed were Damazine town (34°.35'E, 11°.82' N), Guneess (34° 40' E, 11° 80 ' N) and Al Rosseires (34° 38'E, 11° 80' N)], White Nile [site surveyed were Kosti (32°.67' E, 13°.14' N), Rabak (32°.70' E, 13°.13' N) and Asalaya (32°.73' E, 13°.25' N)], Khartoum [site surveyed were Al Rimeilah (32° 31' E, 15° 33' N) and Al Kalaklah (32° 30' E, 15° 32' N)] and Gadaref state [sites surveyed were Gadaref town (34° 16' E, 14° 04' N), Al Faw (35° 38' E, 14° 19' N) and Al Shuwak (35° 85' E, 14° 42' N)] (Figure 1). Because the distance between the sites surveyed within the states is shorter (less than 10 km), each state was treated as one population except Sennar and Gadaref where Mayirno, Al Faw and Al Shuwak were considered different populations due to the relatively long distances (i.e. the distance between Al Faw and Al Shuwak is more than 180 km) which separate them (Figure 1). These states represent the major malaria endemic areas in eastern and central Sudan. The ITN coverage achieved in 2010 was 94%, 87%, 80%, 77% and 52% in Sennar, Blue Nile, Gadaref, White Nile and Khartoum states, respectively (National Malaria Control Programme, unpublished data). There is no routine IRS programme in these regions although IRS with pyrethroids (e.g. Deltamethrin) is occasionally applied in the rainy season (July - October). DDT has not been used for IRS since 1996 when the last round was done in a remote area.

### Mosquito collections

*Anopheles *larvae were collected based on cross sectional surveys from their natural breeding sites such as animal hoof prints, leakage of pipes, ponds and puddles during July - November 2009. To reduce in-breeding bias, larvae were sampled from more than one (usually at least three) breeding sites. In some states, e.g. Sennar and White Nile, the larvae were collected on two separate occasions over a period of two weeks. The mosquitoes were reared to adulthood, in field insectaries in the major towns of each state and identified using morphological keys [18]. Based on the results from the previous studies in central and eastern Sudan, all insects were treated as *An. arabiensis *as this vector was the only member of the *An. gambiae *complex found in the region [9,10,19,20].

### Insecticide susceptibility tests

Insecticide susceptibility tests were carried out using the standard WHO protocol [21]. Two to three day-old non blood-fed adult female *An. arabiensis *were tested. Batches of 20-25 mosquitoes were exposed to test papers impregnated with 0.75% permethrin or 4% DDT. Controls included batches of mosquitoes from each site exposed to untreated papers. The knockdown effect of each insecticide was recorded every 10 minutes over the one-hour exposure period. Mosquitoes were then transferred to a recovery tube and provided with 10% sugar solution. Final mortality was recorded 24 hours post-exposure.

### Mosquito DNA preparation and kdr genotyping

DNA from a single female mosquito was extracted using the Livak method [22] and resuspended in 50- μL of ddH_2_O. The purity and concentration of DNA were measured using Nanodrop spectrophotometer (Nanodrop ND-1000 Technologies).

Populations from Damazin, Kosti, Sennar and Mayirno were genotyped at the Liverpool School of Tropical Medicine (LSTM) using the Taqman probe described by Bass *et al. *[23]. A small number of samples from Khartoum and Al Shuwak were sequenced.

### Data analysis

Data were analysed using descriptive and inferential statistics. Fifty and ninety five percent knockdown times (KDT_50 _and KDT_90_) were computed using survival probit analysis (AnalystSoft Inc., BioStat v2009). Tukey-Kramer HSD tests were used to determine the difference in the means of mosquito mortality rates between the populations for each insecticide treatment. Chi-square tests were used to compare the frequencies of kdr alleles between the two phenotypes of surviving and dead mosquitoes for each insecticide. The association between the presence (yes/no) of kdr genotype and resistance phenotype (resistance/susceptible) was further confirmed for both insecticides using nominal logistic regression model. This analysis was conducted using JMP statistical software (JMP SAS Institute Inc. 2003).

## Results

### Mortality rates

The mortalities at 24 hours post-exposure are shown in Table 1 and 2. Based on WHO criteria, all populations would be defined as resistant or 'potentially resistant' to permethrin and DDT. A high frequency of peremthrin resistant individuals was found in Kosti (60% mortality), Sennar (61% mortality;) and Damazin (77% mortality). These same three populations, in addition to Mayirno and Al Shuwak populations, showed less than 80% mortality to DDT and are thus defined as resistant (Table 2). The population from Khartoum demonstrated potential resistance to DDT (94.5% mortality (Table 2).

**Table 1:** Mean mortalities and 50% and 90% knockdown times (in minutes) (KDT_50 _and KDT_90_) of female *Anopheles arabiensis *in populations from eastern and central Sudan following exposure to permethrin.

Population	**No**.	Mortalities % (95% CI)	KDT_50 _(95% CI)	KDT_90 _(95% CI)
Sennar	300	61.3 (44.4 - 78.3) ^bc^	31.9 (30.2 - 33.7)	105.1 (91.4 - 118.8)
Mayirno	100	81.0 (79.8 - 82.2) ^abc^	25.3 (18.4 - 32.2)	59.7 (52.7 - 66.6)
Damazin	300	77.3 (60.4 - 94.3) ^abc^	37.4 (29.6 - 43.6)	51.6 (30.3 - 72.8)
Khartoum	175	97.5 (96.3 - 98.7) ^a^	19.8 (18.2 - 21.2)	33.9 (32.1 - 36.2)
Kosti	300	60.0 (43.1 - 77.0) ^c ^	27.9 (21.1 - 51.5)	79.7 (63.8 - 95.5)
Al Shuwak	160	95.6 (94.4 - 96.8) ^ab^	31.0 (26.2 - 35.9)	55.2 (50.3 - 60.1)
Al Faw	100	97.0 (95.8 - 98.2) ^a^	30.1 (24.9 - 35.3)	53.5 (48.3 - 58.7)

Columns not connected by the same letter are significantly different at a level of *P <*0.05 (Tukey-Kramer HSD test).

**Table 2:** Mean mortality of and 50% and 90% knockdown times (in minutes) (KDT_50 _and KDT_90_) of female *Anopheles arabiensis *in populations from eastern and central Sudan in response to DDT exposure.

Population	**No**.	Mortalities % (95% CI)	KDT_50 _(95% CI)	KDT_90 _(95% CI)
Sennar	320	39.7 (19.8 - 59.53)^b^	42.3 (22.5 - 62.2)	77.1 (25.89 - 128.3)
Mayirno	110	49.0 (47.8 - 50.18)^ab^	62.8 (43.5 - 82.2)	97.8 (78.4 - 117.1)
Damazin	300	39.0 (19.1 - 58.86)^b^	45.7 (34.5 - 70.0)	79.5 (71.2 - 93.1)
Khartoum	200	94.5 (93.3 - 95.68)^a^	30.4 (26.8 - 33.8)	53.9 (47.2 - 65.7)
Kosti	300	73.3 (53.5 - 93.2)^ab^	73.8 (70.3 - 77.3)	102.7 (32.8 - 172.7)
Al Shuwak	140	67.1 (66.0 - 68.32)^ab^	60.4 (35.5 - 85.3)	98.2 (73.8 - 123.1)

Columns not connected by the same letter are significantly different at a level of *P <*0.05 (Tukey-Kramer HSD test).

### Knockdown effect

The 50% and 90% knockdown time thresholds (KDT_50 _and KDT_90_) determined over a one-hour period against permethrin and DDT are shown in Table 1 and 2. All populations, with the exception of Khartoum, had similar KDT50s for permethrin. The Khartoum population was knocked down significantly faster with permethrin. Similarly, the Khartoum population had a significantly lower KDT50 with DDT than the other populations. For DDT, the Kosti population took significantly longer to be knocked down than any other population.

### Knockdown resistance (kdr) alleles

Table 3 summarises the presence of 1014F-kdr allele in six populations of *An. arabiensis*. The data are stratified according to whether they survived or died after exposure to the WHO diagnostic dose of permethrin and DDT. Of 248 mosquito specimens screened for both kdr alleles, the 1014F-kdr allele was present in 165 (96 alive and 69 dead) specimens. The 1014S-kdr allele was not detected in any genotyped mosquito specimens. Data from all sites were pooled by insecticide and the correlation between genotype and phenotype was determined. Homozygous 1014F individuals were not more likely to survive (53.7%) permethrin exposure than to be killed (38.6%) by the diagnostic dose (*χ2 *= 2.222, *P *= 0.1361). There was no difference in likelihood of permethrin survival in heterozygotes (*χ2 *= 0.066, d.f. = 1, *P *= 0.7973). The susceptible genotypes were more likely to be killed by permethrin exposure than to survive (*χ2 *= 3.981, *P *= 0.0460) (Table 4). However, 16.7% (9/54) of the permethrin survivors were 1014L homozygotes. The 1014F allele failed to confer a survival advantage to the WHO diagnostic dose of DDT in either the homozygous or heterozygous state. Surprisingly, the heterozygous 1014F individuals were significantly (41.7%) more likely to be killed by DDT than to survive (24.4%) by the diagnostic dose (*χ2 *= 4.913, *P *= 0.0267). Similarly there was no increased risk of DDT-induced death for the 1014L genotype (*χ2 *= 1.521, *P *= 0.2174) and indeed approximately half (43%) the DDT survivors lacked any kdr allele (Table 4). Overall, unlike DDT insecticide (*χ2 *= 1.521, *P *= 0.2174), the nominal logistic regression confirmed a significant association between the present (yes/no) of kdr mutation (L1014F) and permethrin resistance phenotype (resistance/susceptible) (*χ2 *= 3.981, *P *= 0.0460). This could be further supported by the interesting fact that the highest 1014F frequency was observed in the most ever resistant population to permethrin from Kosti (Table 1 &3).

**Table 3:** Summary of 1014F kdr allele in alive and dead mosquitoes of *An. arabiensis *exposed to permethrin and DDT among six populations from eastern and central Sudan.

Origin			kdr genotype				Frequency	
	**Insecticide**	**Phenotype**	**Leu Leu**	**Leu Phe**	**Phe Phe**	**Total**	**S**	**R**

Mayirno	*DDT*	Dead	10	4	4	18	0.67	0.33
		Alive	27	0	1	28	0.96	0.04
	*Permethrin*	Dead	5	3	0	8	0.81	0.19
		Alive	7	0	0	7	1.00	0.00
Sennar	*DDT*	Dead	6	1	0	7	0.93	0.07
		Alive	8	13	4	25	0.58	0.42
	*Permethrin*	Dead	7	0	0	7	1.00	0.00
		Alive	2	12	9	23	0.35	0.65
Kosti	*DDT*	Dead	0	4	11	15	0.13	0.87
		Alive	0	0	15	15	0.00	1.00
	*Permethrin*	Dead	0	7	17	24	0.15	0.85
		Alive	0	4	20	24	0.08	0.92
Damazin	*DDT*	Dead	3	16	0	19	0.58	0.42
		Alive	4	8	8	20	0.40	0.60
Al Shuwak*	*DDT*	Dead	1	0	0	1	1.00	0.00
		Alive	0	0	1	1	0.00	1.00
	*Permethrin*	Dead	2	2	0	4	0.75	0.25
Khartoum*	*DDT*	Alive	0	1	0	1	0.50	0.50
	*Permethrin*	Dead	1	0	0	1	1.00	0.00

*DNA sequence results

**Table 4:** Frequency of 1014F kdr allele in survival and dead mosquitoes of *An. arabiensis *exposed to a WHO discriminating dose of permethrin and DDT.

Insecticide	Phenotype	No. tested	Leu Leu	Leu Phe	Phe Phe
Permethrin	Dead	44	0.34(15)	0.27 (12)	0.39 (17)
	Alive	54	0.17 (9)	0.30 (16)	0.54 (29)
	*P value*		0.0460	0.7971	0.1361
DDT	Dead	60	0.33 (20)	0.42 (25)	0.25 (15)
	Alive	90	0.43 (39)	0.24 (22)	0.32 (29)
	*P value*		0.2174	0.0267	0.3384

## Discussion

Based on the WHO criteria for characterizing insecticide resistance/susceptibility, no evidence for full susceptibility to permethrin or DDT was found among the populations tested. The populations of *An. arabiensis *from Kosti, Sennar and Damazin were resistant to both permethrin and DDT. In addition, resistance to DDT was demonstrated in Mayirno and Al Shuwak. The KDT_50 _and KDT_90 _for DDT in the current study was much higher than those reported for a completely susceptible population from New Halfa, eastern Sudan [9]. DDT was banned in Sudan for agricultural use in 1980 but continued to be used in vector control for a further 15 years. Hence the high level of DDT resistance may be a result of past use in vector control. Other previous studies also showed that the selection of resistance to DDT in populations of malaria vectors was due to the long-standing and extensive use of DDT in the IRS programmes [24,25]. Interestingly the kdr genotype did not correlate well with resistance to DDT with over 43% the survivors being wild type for the kdr allele. This suggests that alternative resistance mechanisms are responsible for the DDT resistance.

Permethrin resistance is now well established in central Sudan with populations of *An. arabiensis *from three states (White Nile, Sennar and Blue Nile) showing less than 80% mortality to permethrin. The frequency of resistance appears to have increased considerably over the past three years as an earlier study in Sennar State found only one of four populations to be resistant to permethrin and none to DDT [10]. In contrast to DDT, permethrin resistance correlates with the presence of the 1014F genotype. However, this association between the presence of kdr and susceptibility/resistance to permethrin could be attributed mainly to the significant presence of the wild-type (Leu Leu) genotype among the dead individuals against the discriminated dose of this insecticide (Table 4). This suggests that this genotype is more likely to associate with susceptible phenotype than resistance in the mosquito vector *An. arabiensis *from Sudan.

Overall, it is interesting to note that the association between the presence of kdr and resistance phenotype was weak for permethrin and absent for DDT, indicating that kdr is a dubious marker of both resistance to these insecticides and to be evidence for control failure in the populations tested of *An. arabiensis*. This association was shown quite strong in the closely related malaria vector *Anopheles gambiae *and it emphasized that kdr genotype might explain only a portion of heritable variation in resistance phenotype and that diagnostic assays to test the importance of other resistance mechanisms in field populations are required [26-28]. This could be further supported by the fact that, in the present study, approximately 16.7% of the survivors *An. arabienis *against permethrin were kdr negative indicating a role for additional pyrethroid resistance mechanisms.

The 1014S mutation was not detected in the six populations screened. The 1014S mutation has previously been observed in one out of three populations in Kassala state [9] but absent in Gezira and Sennar [10]. The result suggests limited distribution of 1014S allele which so far reported only from Kassala town.

## Conclusion

The observed co-resistance to permethrin coupled with the occurrence of high resistance to DDT and high kdr frequency in populations of *An. arabiensis *could greatly affect the malaria vector control in Sudan. Relying on the use of ITNs alone may not continue to provide adequate control if this trend continues. Thus, the national malaria control program may need to consider additional methods for malaria vector control in Sudan.

## Competing interests

The authors declare that they have no competing interests.

## Authors' contributions

YEH supervised field work, performed data management and statistical analyses, provide results interpretation and drafted the manuscript, CMJ and MMA carried out the laboratory analyses, HR supervised the laboratory work, drafted and critically reviewed and finalized this paper for publication. All authors read and approved the final manuscript.
